# Prediction of drug interaction between oral adsorbent AST-120 and concomitant drugs based on the in vitro dissolution and in vivo absorption behavior of the drugs

**DOI:** 10.1007/s00228-016-2102-5

**Published:** 2016-08-05

**Authors:** Yohei Koya, Shinya Uchida, Yoshiki Machi, Yuko Shobu, Noriyuki Namiki, Tsutomu Kotegawa

**Affiliations:** 1 Pharmaceutical & Agrochemicals Division, KUREHA CORPORATION, 3-26-2 Hyakunin-cho, Shinjuku-ku, Tokyo, 169-8503 Japan; 2 Department of Pharmacy Practice and Science, School of Pharmaceutical Sciences, University of Shizuoka, 52-1 Yada, Suruga-ku, Shizuoka, 422-8526 Japan; 3 Oasis Daiichi Hospital, 3-3-19 Higashi-tsurusaki, Oita, 870-0103 Japan

**Keywords:** AST-120, Indoxyl sulfate, Drug interaction, Dissolution, Pharmacokinetics, Dosing interval

## Abstract

**Purpose:**

AST-120 is used to decrease the abundance of serum uremic toxins in treatment of chronic kidney disease; however, it could also adsorb concomitantly administered drugs. This study aimed to develop a prediction method for drug interaction between AST-120 and concomitantly administered drugs based on in vitro dissolution and in vivo absorption behavior.

**Methods:**

Sixty-eight drugs were selected for the analysis. For each drug, theoretical dissolution (*R*_d_) and absorption (*R*_a_) rates at estimated dosing intervals (1, 30, 60, 90, 120, and 240 min) were calculated using the Noyes-Whitney formula and compartment analysis, respectively. The optimal thresholds for *R*_d_ and *R*_a_ (*R*_dth_ and *R*_ath_) were estimated by comparing the results with those of previous drug interaction studies for six drugs. Four drug interaction risk categories for 68 drugs at each dose interval were defined according to the indices of dissolution and absorption against their thresholds.

**Results:**

The in vitro dissolution and in vivo absorption behavior of the selected drugs were well fitted to the Noyes-Whitney formula and one- or two-compartment models. The optimal *R*_dth_ and *R*_ath_ that gave the highest value of consistency with the equivalence of drug interaction studies were 90 and 30 %, respectively. As the dosing intervals were lengthened, the number of drugs classified into the low-risk categories increased.

**Conclusion:**

A new drug interaction prediction method based on the pharmacokinetic parameters of drugs was developed. The new model is useful for estimating the risk of drug interaction in clinical practice when AST-120 is used in combination with other drugs.

**Electronic supplementary material:**

The online version of this article (doi:10.1007/s00228-016-2102-5) contains supplementary material, which is available to authorized users.

## Introduction

Chronic kidney disease (CKD) is a global health problem that is commonly treated with many different medications. As the glomerular filtration rate decreases during the progression of CKD, levels of uremic toxins, such as indoxyl sulfate, increase. Accumulation of uremic toxins appears to contribute to the progression of CKD and cardiovascular disease [1]. AST-120, an orally administered spherical carbon, decreases serum uremic toxins by adsorbing them and their precursors within the gastrointestinal tract [2]. The product of AST-120, Kremezin® (KUREHA CORPORATION, Japan) was approved as a drug in Japan in 1991, and subsequently in Korea and the Philippines, for use in improving uremia symptoms and delaying the initiation of hemodialysis in patients with CKD [3, 4].

AST-120 is a non-specific adsorbent that adsorbs organic substances with molecular weight less than 1000 [5]. Therefore, AST-120 may adsorb concomitantly administered drugs, decreasing their efficacy. Several studies that have examined drug interaction between AST-120 and concomitant drugs in healthy subjects have reported that AST-120 significantly affected the pharmacokinetics of drugs that were administered simultaneously. However, certain dosing intervals have been reported to avoid drug interaction between AST-120 and some concomitantly administered drugs [6–10]. Significant changes in pharmacokinetic parameters were observed for metoprolol extended-release tablets, even when they were administered at a 60-min dosing interval following AST-120 administration [11]. Therefore, the dosing interval allowing the interaction between AST-120 and concomitantly administered medications to be avoided may depend on the characteristics of the drug and/or formulation. Previous drug interaction studies did not evaluate the potential effects of altered dosing intervals on the effectiveness of CKD treatment. In clinical practice, no serious adverse events related to drug interaction between AST-120 and concomitant drugs have been reported. However, given the wide variety of drugs used in the treatment of CKD and the inevitability of the introduction of new medications and formulations in the future, prediction of drug interaction between AST-120 and concomitant drugs based on their characteristics and formulation types may guide clinicians regarding CKD treatment and advice given to CKD patients regarding drug administration.

Two studies have investigated drug interaction between oral adsorbents and concomitant drugs in vitro [12, 13]. Although these studies provide useful information on drug interaction between oral adsorbents and concomitantly administered drugs, their clinical use is limited because the dissolution behavior and in vivo absorption behavior of the drugs were not considered.

The objective of this study was to develop a prediction method for drug interaction based on the dissolution behavior and absorption behavior of drugs concomitantly administered with AST-120 in CKD treatment. In addition, the risk categories of concomitantly administered drugs were defined using the prediction method.

## Materials and methods

### Data sources

Drugs that were concomitantly administered with AST-120 in CKD treatment in 2009 in Japan were surveyed, and 68 drugs, including those administered to patients with CKD or those whose interaction with AST-120 has been evaluated, were selected for the analysis. Information regarding dissolution tests and pharmacokinetics in humans was obtained from the Japanese Orange Book [14], package inserts, and the literature. The doses of the formulations used in the dissolution test and the doses used in the pharmacokinetic study were determined according to the following rules: (a) the same dose was selected for the dissolution test and pharmacokinetic study; (b) the maximum dose in standard clinical practice in Japan was selected; (c) when drug interaction study results were available, the dose used in the study was selected; (d) when data conforming to rules (a), (b), and (c) were unavailable in the public domain, the data for the largest dose reported in the public domain was selected. The pharmacokinetic data used for the calculation of *k*_a_ and *T*_laga_ were obtained from healthy subjects under fasting conditions, except for data regarding the metoprolol extended-release tablet.

### Calculation of parameters for drug dissolution from the dissolution test

The dissolution rate constant (*k*_d_) values were calculated according to the following procedure. The dissolution rates with time were read from the dissolution test graphs with the first fluid (pH 1.2) and the second fluid (pH 6.8) for the dissolution test (the 16th edition of the Japanese Pharmacopoeia), and defined as measured values. The dissolution rates were calculated by the extended Eq. (2) of the Noyes-Whitney formula (1) and recorded as estimated values (*R*_d_). Next, *k*_d_ values where the residual sum of squares of the measured and estimated values at each sampling points were the lowest were determined using the solver function of Microsoft® Office Excel 2010. When lag time existed in the dissolution process, such as for encapsulated formulations and enteric-coated formulations, the time for 1 % dissolution was defined as the lag time (*T*_lagd_), and the *k*_d_ value was calculated by the Eq. (3).

Data were excluded from the analysis when complete dissolution was not achieved in both of the fluids during the test. When *k*_d_ values were calculated from both the first fluid and the second fluid for the Japanese Pharmacopoeia dissolution test, the higher value was used for drug interaction prediction. When complete dissolution was achieved in only one of the fluids, the obtained value was used for drug interaction prediction. When *R*_d_ was less than 0 %, it was recorded as 0 %.

1 $$ \frac{dC}{dt}=kS\left(C{}_{\mathrm{s}}-C\right) $$

2 $$ \begin{array}{l}{k}_{\mathrm{d}}=kS\hfill \\ {}C={C}_{\mathrm{s}}\kern0.15em \left\{1- \exp \left(-{k}_{\mathrm{d}}t\right)\right\}\hfill \\ {}{R}_{\mathrm{d}}\kern0.1em \left(\%\right)={x}_{\mathrm{e}}\kern0.15em \left\{1- \exp \left(-{k}_{\mathrm{d}}t\right)\right\}\hfill \end{array} $$

3 $$ \mathrm{When}\ \mathrm{lag}\ \mathrm{time}\ \mathrm{exists},\kern0.5em {R}_{\mathrm{d}}\kern0.15em \left(\%\right)={x}_{\mathrm{e}}\kern0.15em \left[1- \exp \left\{-{k}_{\mathrm{d}}\;\left(t-{T}_{\mathrm{lagd}}\right)\right\}\right] $$

(*C*, concentration in the fluid; *C*_s_, saturating concentration; *k*, apparent rate constant; *S*, surface area of the formulation; *k*_d,_ dissolution rate constant; *R*_d_, dissolution rate; *x*_e,_ dissolution rate in an equilibrium state. The calculation was made only when *x*_e_ reached 100 %).

### Calculation of the parameters for in vivo drug absorption from the pharmacokinetic study

The parameters for the absorption rate (*R*_a_) were calculated according to the following procedure. The plasma concentrations of drugs after oral administration were read from the graph at each sampling point; after which, pharmacokinetic parameters were estimated using WinNonlin® (version 6.3, Certara USA Inc., USA). The one- or two-compartment model providing the best fit to the plasma concentration data was chosen visually. Next, *R*_a_ (%) values, which represent the percentage of the maximum absorption at particular time points (*t*) after the administration, were calculated from absorption rate constant (*k*_a_) and lag time (*T*_laga_), which were estimated from the pharmacokinetic data using Eq. (4). When *T*_laga_ was less than 0, it was recorded as 0. When *R*_a_ was less than 0 %, it was recorded as 0 %.

4 $$ {R}_{\mathrm{a}}\left(\%\right)=100\ \left[1- \exp \left\{-{k}_{\mathrm{a}}\left(t-{T}_{\mathrm{laga}}\right)\right\}\right] $$

### Evaluation of the predictability of the drug interaction prediction method

When AST-120 and concomitant drugs are administered at an interval (concomitant drugs are administered first), the dissolution and absorption behavior of the concomitant drugs were the same as those in a single-drug condition (non-concomitant administration) until the time of AST-120 administration, because there was no contact between AST-120 and the drugs during that time. If *R*_d_ and/or *R*_a_ are sufficiently high, concomitantly administered drugs will be absorbed completely before they contact AST-120, avoiding drug interaction. Therefore, we estimated the thresholds of *R*_d_ and *R*_a_ (*R*_dth_ and *R*_ath_, respectively) that are required to prevent drug interaction. These thresholds allow the equivalence and non-equivalence of the pharmacokinetics of concomitantly administered drugs to be evaluated.

First, *R*_d_ and *R*_a_ at the time of AST-120 administration after concomitant drug administration (dosing interval) in previous drug interaction studies were calculated (Table 1) [6–11]. Drugs that were administered after AST-120 were excluded from the analysis. When AST-120 and concomitantly administered drugs were administered simultaneously, the time (*t*) was set as 1 min for the calculation of *R*_d_ and *R*_a_. *R*_dth_ and *R*_ath_ were defined as follows: if *R*_d_ ≥ *R*_dth_ and *R*_a_ ≥ *R*_ath_, the predictions were regarded as equivalent.

**Table 1:** Information from previous human drug interaction studies

Drugs	Dosing interval (time before administration of AST-120)^a^	Experimental condition				Change of AUC by concurrent administration of AST-120^b^	Change of C_max_compared with administration alone^b^	Evaluation of equivalence^c^	Reference no.
		Subject	Experimental design	Number of subjects	Dose				
Amlodipine	Simultaneous	Healthy subjects	Modified cross-over design	8	5 mg	NS	16 %↓	NE	6
	30 min					NS	11 %↓	NE	
	90 min					NS	NS	E	
	240 min					NS	NS	E	
Aspirin, dihydroxyaluminum aminoacetate, magnesium carbonate (Bufferin combination tablet)	Simultaneous	Healthy subjects	Unknown	5	810 mg	34 %↓	36 %↓	NE	7
	60 min					NS	NS	E	
Losartan	30 min	Healthy subjects	Cross-over design	30	100 mg	NS	NS	NE	8
						Active metabolite NS	Active metabolite 17 %↓		
	60 min					NS	NS	E	
						Active metabolite NS	Active metabolite NS		
Metoprolol ER	Simultaneous	Healthy subjects	Cross-over design	34	100 mg	30 %↓	22 %↓	NE	11
	60 min					27 %↓	NS	NE	
Nifedipine	Simultaneous	Healthy subjects	Cross-over design	12	5 mg	NS	NS	E	9
	30 min					NS	NS	E	
	120 min					NS	NS	E	
Triazolam	Simultaneous	Healthy subjects	Cross-over design	12	0.25 mg	41 %↓	33 %↓	NE	10

*NS* not significant, *E* equivalent, *NE* non-equivalent

^a^Under fasting conditions except for the losartan study, in which a high-fat diet was given 30 min before AST-120 administration, and metoprolol ER, in which a breakfast was given 10 min before AST-120 administration

^b^For amlodipine, aspirin, and nifedipine, the difference was evaluated as NS when there was no significant difference compared with the control (administration alone). When there was a significant difference, the percentage of the change was expressed. For losartan, triazolam, and metoprolol extended-release tablets, the parameters after logarithmic transformation were used. The difference was evaluated as NS when the 90 % confidence interval of the ratio of the mean compared to the control was within the range of 80 to 125 %, or the ratio of the mean compared to the control was within 90 to 111 %. When there was a significant difference, the percentage of the change was expressed

^c^The equivalence was regarded as E when there was no significant difference in AUC_t_ (or AUC_∞_) or C_max_. The difference was regarded as NE when any of the parameters was significantly different. For the losartan study in which both the unchanged drug and active metabolite were used, the equivalence was regarded as E only when the differences were evaluated as NS for both compounds

Pharmacokinetic data was obtained from previous studies (14 results of 6 drugs) examining drug interaction between AST-120 and concomitantly administered drugs in healthy subjects (Table 1). The evaluation of the equivalence of pharmacokinetic parameters was performed according to the statistical methods or equivalence evaluation methods used in the original studies (Table 1).

After arbitrarily changing the values of *R*_dth_ and *R*_ath_, the predictions were regarded as equivalent if both *R*_d_ ≥ *R*_dth_ and *R*_a_ ≥ *R*_ath_. Otherwise, when *R*_d_ < *R*_dth_ and *R*_a_ < *R*_ath_, the predictions were regarded as non-equivalent. Consistency was evaluated by comparing the equivalence prediction results with the results of the drug interaction studies. When *R*_d_ < *R*_dth_ and *R*_a_ ≥ *R*_ath_ or *R*_d_ ≥ *R*_dth_ and *R*_a_ < *R*_ath_, the evaluation of the results from the predictions and drug interaction studies was regarded as non-consistent.

The predictive value of equivalence was calculated by Eq. (5) for 14 results regarding 6 drugs.

5 $$ \mathrm{Predictive}\ \mathrm{value}\ \left(\%\right)=\mathrm{number}\ \mathrm{of}\ \mathrm{consistency}/\mathrm{number}\ (14)\ \mathrm{of}\ \mathrm{results}\ \mathrm{in}\ \mathrm{previous}\kern0.5em \mathrm{drug}\ \mathrm{in}\mathrm{teraction}\kern0.5em \mathrm{studies}\times 100 $$

The optimal *R*_dth_ and *R*_ath_ were defined when the maximum predictive value was obtained.

### Grouping of concomitantly administered drugs

Grouping of the 68 concomitantly administered drugs, which were selected from the results of the survey conducted in 2009, was performed using a drug interaction prediction method. *R*_d_ and *R*_a_ values were calculated at 1 min (actually simultaneous), 30 min, 60 min, and 120 min. Four risk categories were defined according to the indices of *R*_d_ and *R*_a_ against their optimal thresholds (*Th*_Rd_ and *Th*_Ra_): group 1, *R*_d_ ≥ *Th*_Rd_ and *R*_a_ ≥ *Th*_Ra_; group 2, *R*_d_ < *Th*_Rd_ and *R*_a_ ≥ *Th*_Ra_; group 3, *R*_d_ ≥ *Th*_Rd_ and *R*_a_ < *Th*_Ra_; and group 4, *R*_d_ < *Th*_Rd_ and *R*_a_ < *Th*_Ra_.

## Results

### Calculation of the parameters for in vitro drug dissolution and in vivo drug absorption

**Fig. 1:**
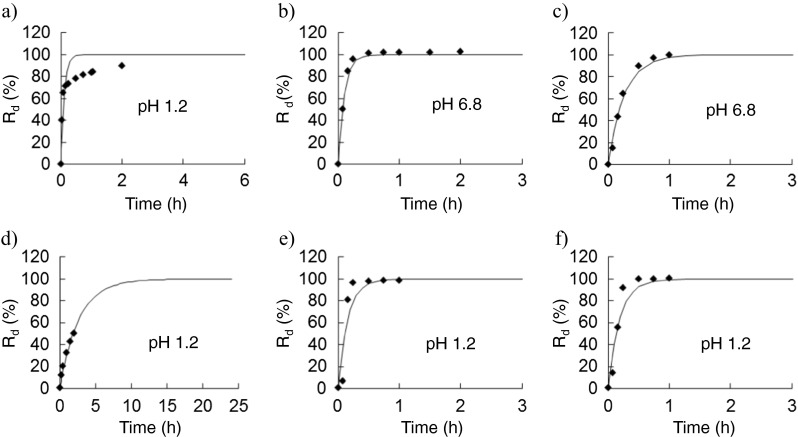
Fitting of the in vitro dissolution behavior of drugs evaluated in drug interaction studies to the Noyes-Whitney formula. **a** Amlodipine. **b** Aspirin, dihydroxyaluminum aminoacetate, and magnesium carbonate (Bufferin combination tablet). **c** Losartan. **d** Metoprolol ER. **e** Nifedipine. **f** Triazolam. Plot: measured value, *line*: estimated value, *R*_d._: dissolution rate. The higher *k*_d_ value was selected from the results of the dissolution test using the first fluid (pH 1.2) and the second fluid (pH 6.8) for the dissolution test (the 16th edition of the Japanese Pharmacopoeia)

Figure 1 shows comparisons of measured and estimated dissolution rates of drugs evaluated in the previous drug interaction studies shown in Table 1 (the comparisons about the other 62 drugs are available as supplemental figure). The results showed good fitting of Eq. (2) or (3) to the measured data.

**Fig. 2:**
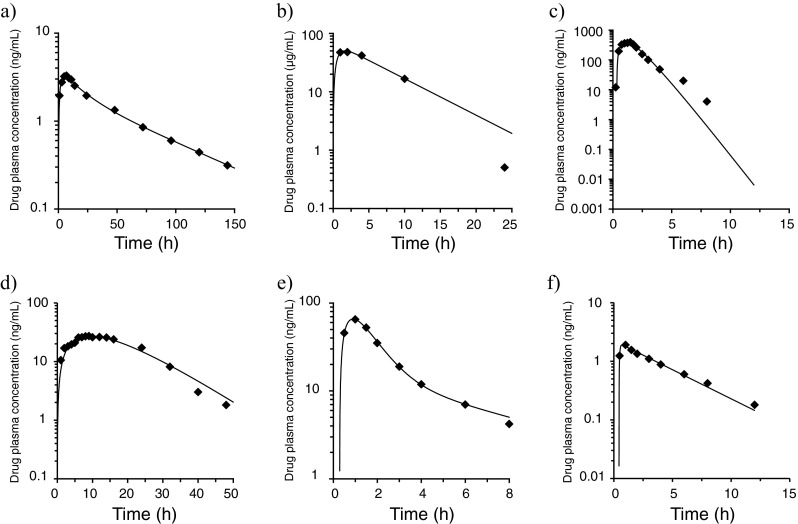
Fitting of the in vivo absorption behavior of drugs evaluated in drug interaction studies using WinNonlin®. **a** Amlodipine. **b** Aspirin, dihydroxyaluminum aminoacetate, and magnesium carbonate (Bufferin combination tablet). **c** Losartan. **d** Metoprolol ER. **e** Nifedipine. f Triazolam. Plot: measured value, *line*: estimated value. The better fitting model is shown

Figure 2 shows comparisons of measured and estimated absorption rates of drugs evaluated in previous drug interaction studies. The results showed good fitting to the optimal model.

Table 2 summarizes the calculated parameters of *k*_d_, *T*_lagd_, *k*_a_, *T*_laga_, *R*_d_, and *R*_a_ for the drugs. *R*_d_ and *R*_a_ were calculated for the dosing interval of each drug (time before administration of AST-120).

**Table 2:** Rate constant parameters of drugs investigated in previous human drug interaction studies

Drugs	Dosing interval (time before administration of AST-120)	*k*_d1.2_ (10^−4^·s^−1^)	*k*_d6.8_ (10^−4^·s^−1^)	*T*_lagd_ (h)	*R*_d_ (%)	*k*_a_ (h^−1^)	*T*_laga_ (h)	*R*_a_ (%)
Amlodipine	Simultaneous	25.54	3.99	0.00	14.2	0.62	0.00	1.0
	30 min				99.0			26.8
	90 min				100.0			60.8
	240 min				100.0			91.8
Aspirin, dihydroxyaluminum aminoacetate, magnesium carbonate (Bufferin combination tablet)	Simultaneous	11.00	27.64	0.00	15.3	1.55	0.00	2.6
	60 min				100.0			78.8
Losartan	30 min	0.6	10.5	0.00	84.8	1.43	0.33	21.7
	60 min				97.7			61.6
Metoprolol ER	Simultaneous	1.03	0.76	0.00	0.6	0.11	0.00	0.2
	60 min				30.9			10.7
Nifedipine	Simultaneous	17.59	11.58	0.00	10.0	2.49	0.28	0.0
	30 min				95.8			41.6
	120 min				99.8			98.6
Triazolam	Simultaneous	14.69	11.08	0.00	8.4	17.34	0.44	0.0

Using the Noyes-Whitney formula, *k*_d1.2_ (dissolution rate constant for the first fluid for the dissolution test, pH 1.2) and k_d6.8_ (dissolution rate constant for the second fluid for the dissolution test, pH 6.8) were calculated. The higher value was used for the prediction. *T*_lagd_ (lag time to 1 % dissolution) and *R*_d._ (dissolution rate at each dosing interval between AST-120 and concomitant drug administration) were calculated. When AST-120 and the concomitantly administered drug were administered simultaneously, the time interval was set as 1 min for the calculation

*k*_a_ (absorption rate constant), *T*_laga_ (lag time in absorption), and *R*_a_ (absorption rate compared to total absorption until the time of AST-120 administration) were calculated using the better-fitted model with WinNonlin® from visual inspection

### Determination of the optimal ranges of *R*_dth_ and *R*_ath_ based on comparisons between the predictions and drug interaction study results

**Fig. 3:**
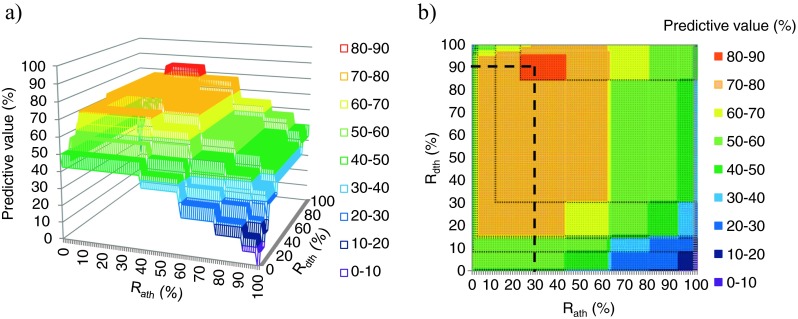
Relationship between threshold and predictive values of *R*_d_ and *R*_a_ The relationships between *R*_ath_ and *R*_dth_ and predictive values for all drugs and all dosing intervals are shown. Predicted values were explored by varying the magnitudes of *R*_dth_ (threshold value of *R*_d_) and *R*_ath_ (threshold value of *R*_a_, the rate relative to the total absorption until the time of AST-120 administration). The prediction was regarded as correct when the results for the equivalence of the prediction and the pharmacokinetic parameters were consistent. **a** Three dimensional graph. **b** Contour graph

The consistency of the equivalence predictions with pharmacokinetic parameters reported in previous drug interaction studies was evaluated. In the case of amlodipine, *R*_d_ and *R*_a_ were 14.2 and 1.0 %, respectively (Table 2), when AST-120 was administered simultaneously with the drug. The prediction was regarded as non-equivalent when *R*_dth_ > 14.2 % and *R*_ath_ > 1.0 %. The result of the drug interaction study was non-equivalent (Table 1); therefore, the prediction was defined as consistent with the result of the drug interaction study when *R*_dth_ > 14.2 % and *R*_ath_ > 1.0 %. Similarly, when the *R*_dth_ and *R*_ath_ values of amlodipine at the 30-, 90-, and 240-min dosing intervals were >99.0 and >26.8 %, ≤100.0 and ≤60.8 %, and ≤100.0 and ≤91.8 %, respectively (Table 2), the predictions were consistent. In the same manner, *R*_dth_ and *R*_ath_ for all drugs and dosing intervals, which showed consistency, were estimated. The predictive values for all drugs and dosing intervals were examined by varying *R*_dth_ and *R*_ath_. Figure 3 shows the relationship between the thresholds and the predictive values. The maximum predictive values were 85.7 % in the range of 84.8 to 95.8 % for *R*_dth_ and 21.7 to 41.6 % for *R*_ath_.

### Grouping of concomitantly administered drugs using *Th*_Rd_ and *Th*_Ra_

**Fig. 4:**
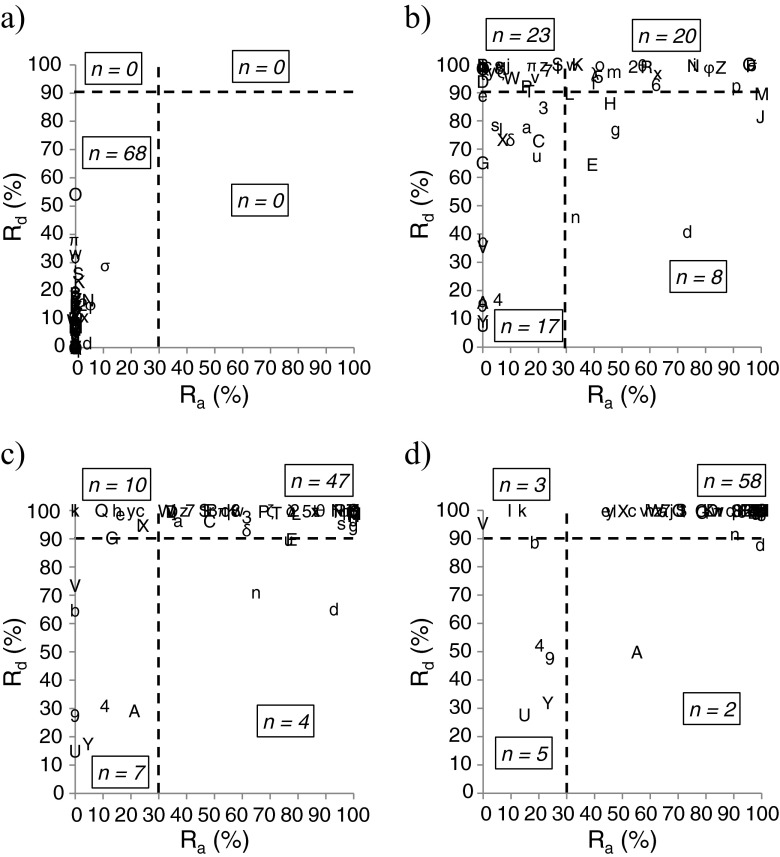
Changes in risk group classification according to dosing interval. *R*_d_: dissolution rate, *R*_a_: absorption rate relative to total absorption until the indicated time point. **a** 1 min (actually simultaneous). **b** 30 min. **c** 60 min. **d** 120 min. *R*_d_ and *R*_a_ were plotted. The number (*n*) of drugs classified into each group when threshold values of *R*_d_ and *R*_a_ were set as 90 and 30 %, respectively, is shown. *1*, amlodipine; *2*, aspirin, dihydroxyaluminum aminoacetate, magnesium carbonate (Bufferin combination tablet); *3*, losartan; *4*, metoprolol ER; *5*, nifedipine; *6*, triazolam; *7*, allopurinol; *8*, ambroxol; *9*, ambroxol ER; *a*, arotinolol; *b*, aspirin (Bayaspirin); *c*, atenolol; *d*, atorvastatin; *e*, azosemide; *f*, bacampicillin; *g*, benidipine; *h*, betaxolol; *i*, brotizolam; *j*, cefcapene pivoxil; *k*, cefdinir; *l*, celiprolol; *m*, cetirizine; *n*, clarithromycin; *o*, clonidine; *p*, clotiazepam; *q*, cyclosporine; *r*, digoxin; *s*, dilazep; *t*, enalapril; *u*, epalrestat; *v*, epinastine; *w*, etizolam; *x*, etodolac; *y*, famotidine; *z*, furosemide; *A*, glibenclamide; *B*, guanabenz; *C*, ibuprofen; *D*, imidapril; *E*, indapamide; *F*, isosorbide; *G*, lansoprazole; *H*, levofloxacin; *I*, lisinopril; *J*, loxoprofen; *K*, metformin; *L*, metoprolol; *M*, naftopidil; *N*, nateglinide; *O*, nicorandil; *P*, nilvadipine; *Q*, omeprazole; *R*, perindopril; *S*, pioglitazone; *T*, pravastatin; *U*, propranolol ER; *V*, rabeprazole; *W*, rebamipide; *X*, sodium ferrous citrate; *Y*, tamsulosin ER; *Z*, temocapril; *δ*, ticlopidine; *ζ*, tizanidine; *θ*, torasemide; *λ*, trandolapril; *π*, trichlormethiazide; *σ*, warfarin; *ϕ*, zolpidem

Figure 4 shows a scatter plot with *R*_d_ and *R*_a_ for 68 drugs concomitantly administered with AST-120 at 1 min (actually simultaneous), 30 min, 60 min, and 120 min. To classify the drugs into different drug interaction risk categories, we set *Th*_Rd_ and *Th*_Ra_ at 90 and 30 %, respectively, from the range of the maximum predictive values (Fig. 3). All drugs were classified into group 4 at a 1-min interval (actually simultaneous). As the dosing interval was lengthened, the values of *R*_a_ and *R*_d_ became greater, while the number of drugs classified into low-risk categories (groups 1 and 2) increased.

## Discussion

In this study, we developed a prediction method for drug interaction based on the in vitro dissolution behavior and in vivo absorption behavior of drugs concomitantly administered with AST-120 in the treatment of CKD. We examined formulas by Noyes-Whitney, Hixson-Crowell, and Higuchi and extended Eqs. (2) and (3) of the Noyes-Whitney formula for the calculation of in vitro dissolution. The Noyes-Whitney formula was better fitted to the measured and estimated values (data not shown). The Noyes-Whitney formula, which is based on the diffusion equation, had good results when a constant surface area of the formulation was assumed, suggesting that many drugs had no significant changes in the surface area of the formulations during drug dissolution. In addition, fitting of the pharmacokinetic data using a one- or two-compartment model was suitable for calculating in vivo absorption.

The relevance of logical prediction of drug interaction based on calculated dissolution and absorption parameters and previous drug interaction studies was examined. Given that previous drug interaction studies investigated pharmacokinetic parameters at different dosing intervals, *R*_d_ and *R*_a_ were calculated in this study at each time point; after which, we estimated the optimal percentages of *R*_d_ and *R*_a_ at the time of AST-120 administration for avoiding drug interaction by examining the prediction values (the consistency between the equivalent/non-equivalent percentages given by the prediction method and the drug interaction study results) by varying *R*_dth_ and *R*_ath_. The results showed that the optimal ranges of *R*_dth_ and *R*_ath_ were 84.8 to 95.8 % and 21.7 to 41.6 %, respectively. The results demonstrated that the prediction value of the drug interaction prediction method was as high as 85.7 %.

Here, we demonstrated that our prediction model has relevance to the drug interaction study results. Subsequently, *R*_d_ and *R*_a_ of 68 drugs concomitantly administered with AST-120 at 1 min (actually simultaneous), 30 min, 60 min, and 120 min were calculated. By comparing these values with the thresholds (*Th*_Rd_ = 90 %, *Th*_Ra_ = 30 %), the drugs were classified into four risk categories. Group 1 was the lowest risk category, where both *R*_d_ and *R*_a_ were above the threshold levels, indicating that both dissolution and absorption were rapid. In group 2, *R*_d_ was below each threshold level, while *R*_a_ was above each threshold level, indicating that dissolution was slow while absorption was rapid, posing a relatively lower risk of adverse effects on the pharmacokinetics and efficacy of drugs in this group. In group 3, *R*_d_ was above each threshold level, while *R*_a_ was below each threshold level, indicating that dissolution was rapid while absorption was slow, posing a risk of adsorption of the active substance to AST-120 before its absorption, which warrants attention. Group 4 was the highest risk category. In this group, both *R*_d_ and *R*_a_ were below each threshold level, indicating slow dissolution and absorption, as well as the greatest risk of drug interaction. All drugs were classified into group 4 at 1-min interval (almost simultaneous). As the dosing interval was lengthened, the values of *R*_a_ and *R*_d_ became greater, while the number of drugs classified into the lowest risk category (groups 1 and 2) increased. For example, allopurinol was classified into group 3 at a 30-min dosing interval, while it was classified into group 1 at dosing intervals of 60 min or longer. These results suggest that the detrimental effects of drug interaction between AST-120 and concomitantly administered drugs on pharmacokinetics and drug efficacy can be reduced by increasing dosing intervals. In addition, ambroxol hydrochloride, an extended-release capsule, was classified into group 4 at all dosing intervals. The preferable dosing interval to avoid the risk of drug interaction varies with the individual drug and is determined by the dissolution and absorption characteristics. Many extended-release formulations were classified into the high-risk categories; therefore, clinicians should pay attention to drug interaction when using such formulations with AST-120.

Two studies have investigated drug interaction between oral adsorbents and concomitantly administered drugs in vitro [12, 13]. Although these studies provide useful information on drug interaction between oral adsorbents and concomitantly administered drugs, their clinical use is limited because the dissolution behavior and absorption behavior of the drugs were not considered. Our present prediction method is a totally new approach because it considered the dissolution and absorption behaviors of concomitantly administered drugs used in clinical practice. Thus, the risk of drug interaction is likely to be avoided by estimating the minimum dosing intervals between AST-120 and concomitantly administered drugs based on our risk classification system and the dissolution and absorption rate thresholds. In addition, this prediction method can be performed using information available in the public domain. Our prediction method is a useful approach for estimating the potential of drug interaction for many drugs that are concomitantly administered with AST-120 in CKD treatment.

A considerable limitation of this study was that our prediction method was not based on original experimental data but on reliable in vitro and in vivo data obtained from third-party sources. Although our prediction method could be useful for clinical pharmacologists and clinicians because it utilizes only information available in the public domain, the new method should be experimentally validated in the future. Moreover, evaluation of the relationship between our prediction method and the other prediction method could also be important for the validation. The adsorbent characteristics of AST-120 were not considered. Considering that the effects of adsorbents on the pharmacokinetics of concomitantly administered drugs are small when the amount/rate of adsorption is small, a classification system incorporating factors related to adsorbents may be more useful. When a drug has poor solubility, but a high *k*_d_ value (*k*_d_ refers to the time required to reach dissolution equilibrium), prediction of drug interaction is difficult. Classical physicochemical properties such as molecular mass, water solubility, logP/D, and dissociation constant (pKa) might be useful to modify our prediction method. Furthermore, the validity of the prediction method in this study remains to be confirmed, because our method was based on a limited number of drug interaction study results. Further drug interaction studies are required to improve the accuracy of prediction.

The present study provides a newly developed drug interaction prediction method based on the pharmacokinetic parameters of drugs. Although monitoring of the blood concentration and effectiveness of concomitantly administered drugs is required for accurate predictions, drug interaction prediction based on the characteristics of drugs and formulations is useful for estimating the risk of drug interaction in clinical practice when AST-120 is used in combination with other drugs.

## Electronic supplementary material

ESM 1 (PDF 312 KB)
