# Supplementary material for: Prediction of drug interaction between oral adsorbent AST-120 and concomitant drugs based on the in vitro dissolution and in vivo absorption behavior of the drugs
Source: Eur J Clin Pharmacol. 2016 Aug 5;72(11):1353–61. doi: 10.1007/s00228-016-2102-5 (PMC5055906; doi:10.1007/s00228-016-2102-5)
Supplement: Supplementary file 1 — (PDF 312 KB) [file 228_2016_2102_MOESM1_ESM.pdf]

Fitting of the in vitro dissolution behavior of drugs to the Noyes-Whitney formula

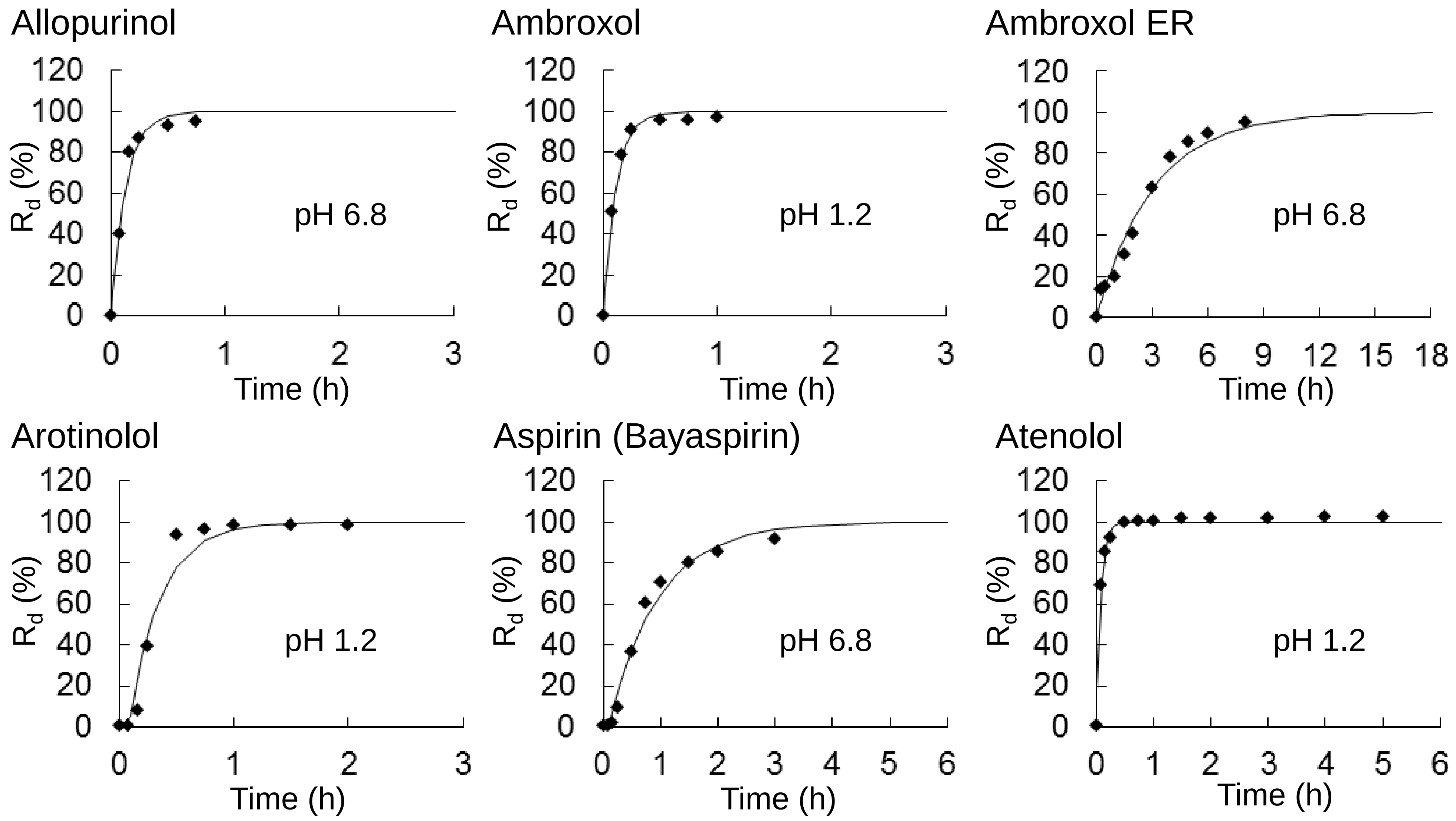

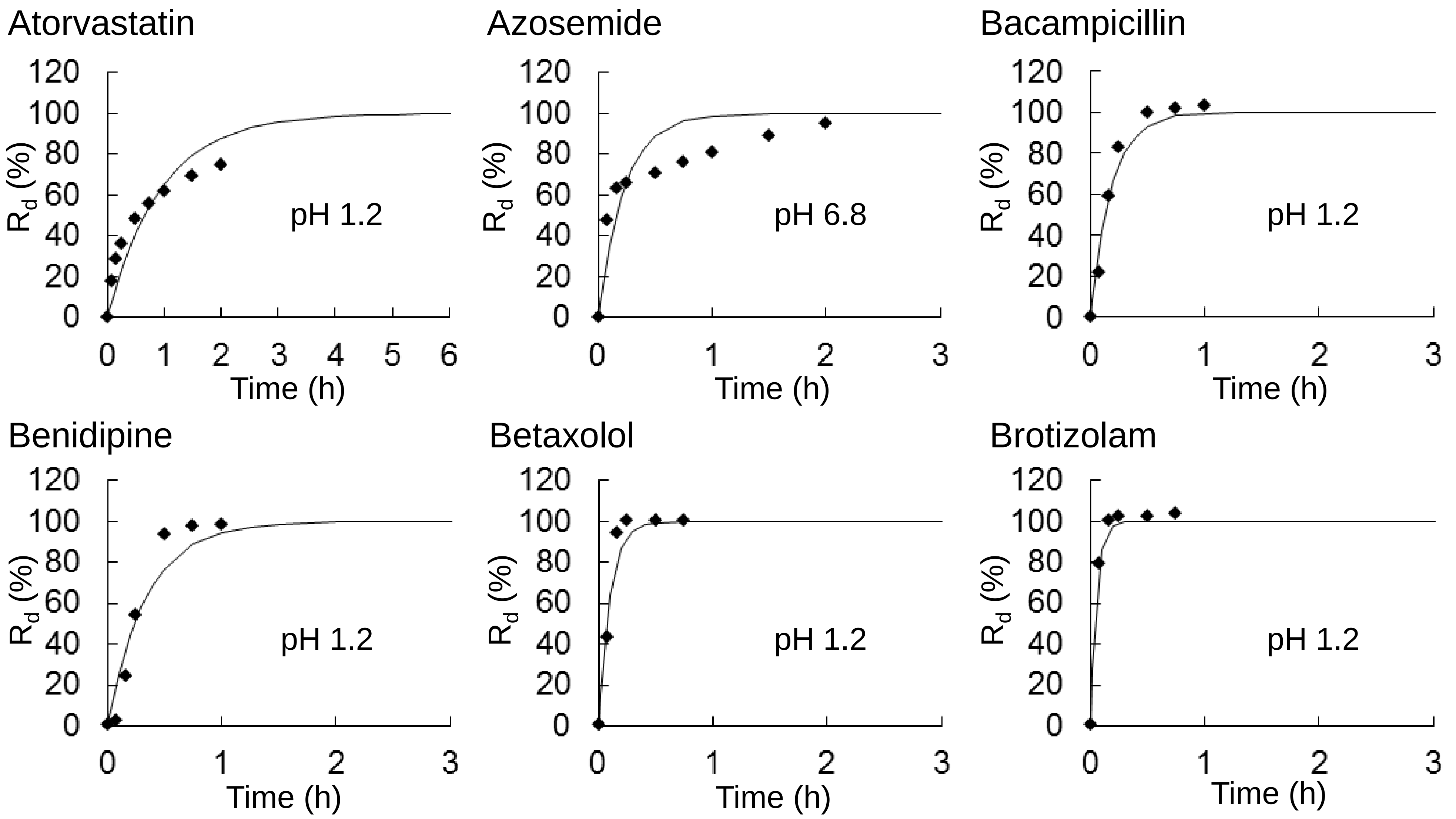

Atorvastatin
pH 1.2
Azosemide
pH 6.8
Bacampicillin
pH 1.2
Benidipine
pH 1.2
Betaxolol
pH 1.2
Brotizolam
pH 1.2
R_d (%)
Time (h)
0
20
40
60
80
100
120
0
1
2
3
4
5
6

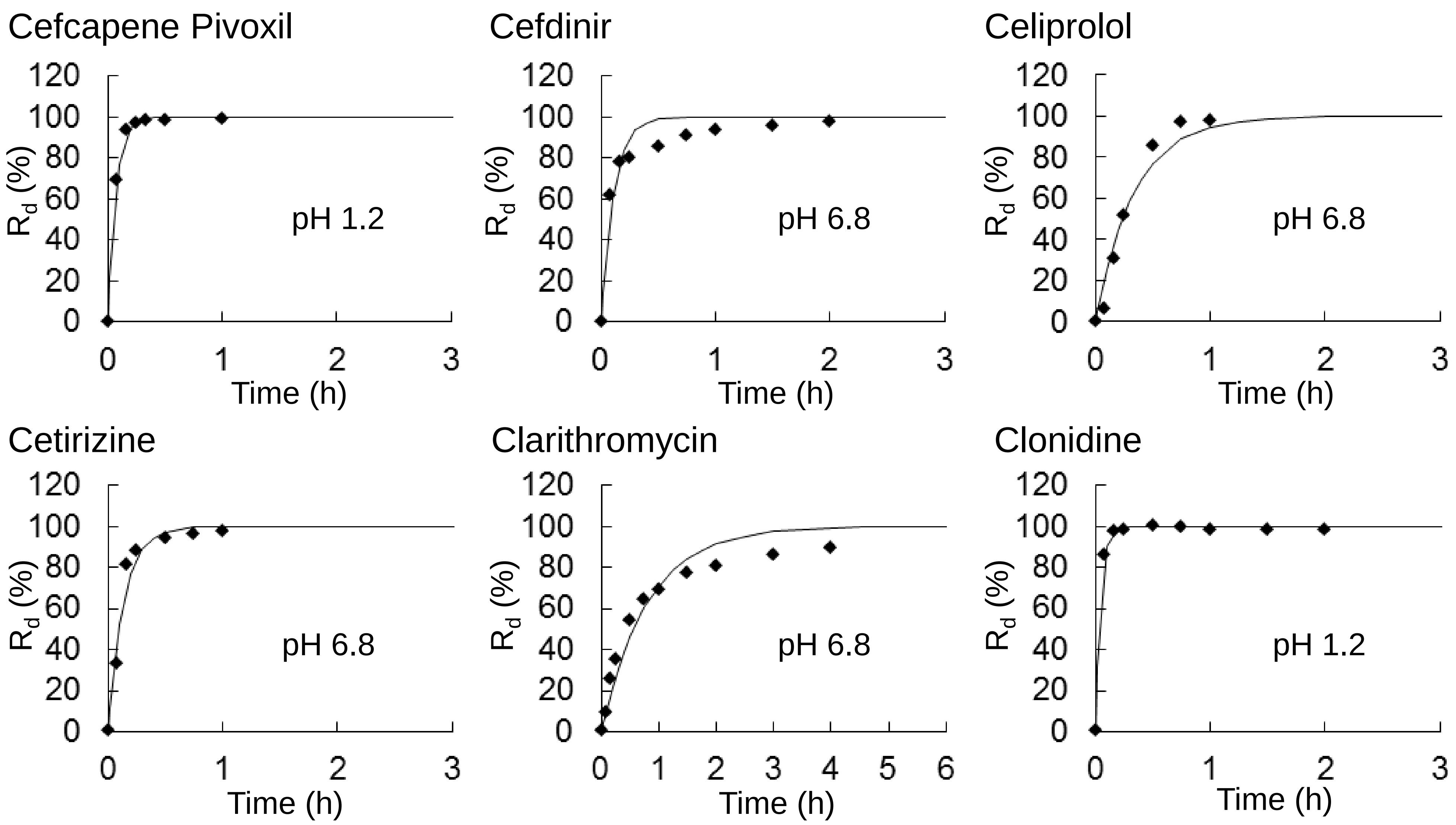
Cefcapene Pivoxil
pH 1.2
Cefdinir
pH 6.8
Celiprolol
pH 6.8
Cetirizine
pH 6.8
Clarithromycin
pH 6.8
Clonidine
pH 1.2
R_d (%)
Time (h)

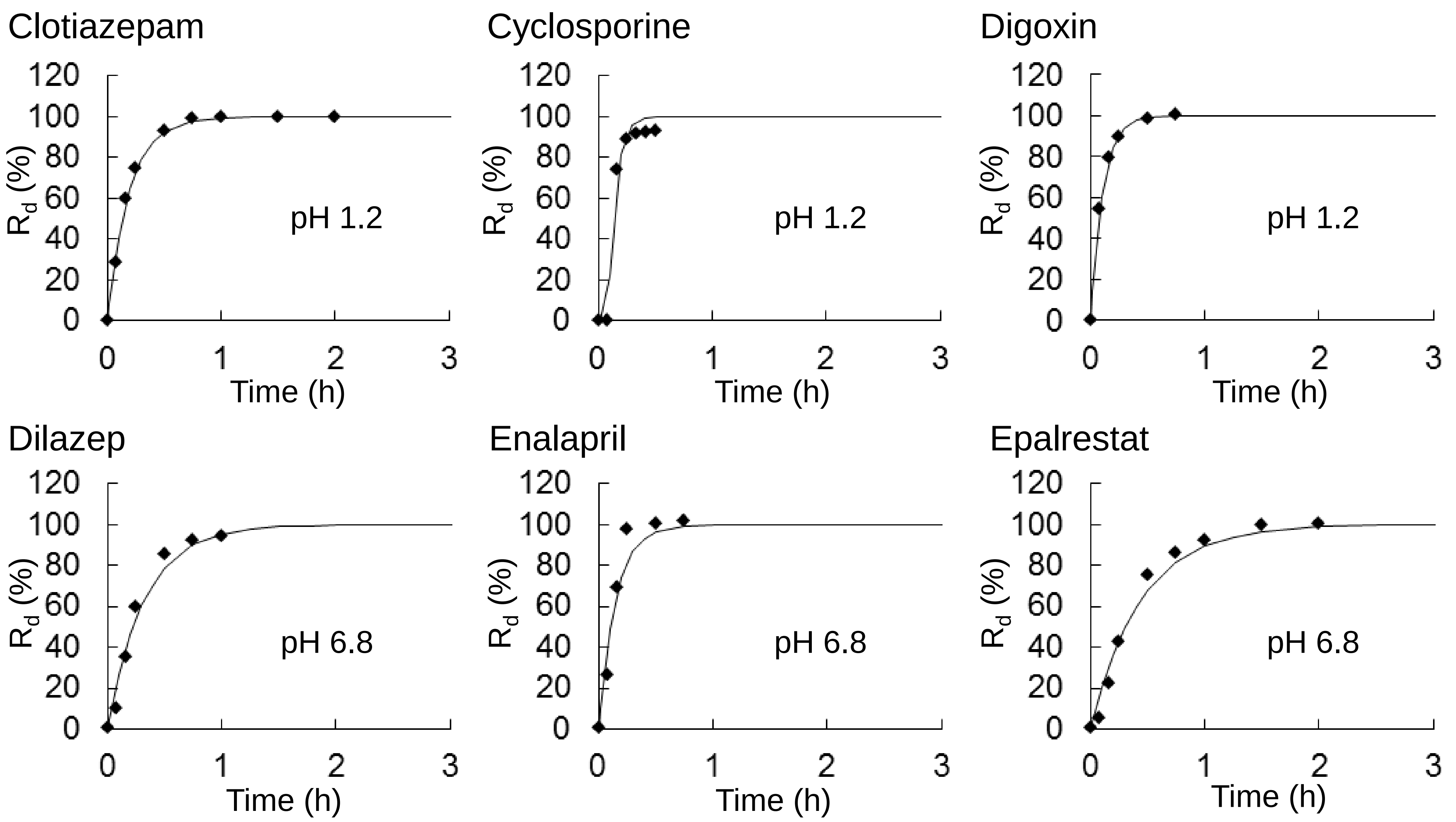
Clotiazepam
Cyclosporine
Digoxin
Dilazep
Enalapril
Epalrestat
pH 1.2
pH 6.8
R_d (%)
Time (h)
0
20
40
60
80
100
120
1
2
3

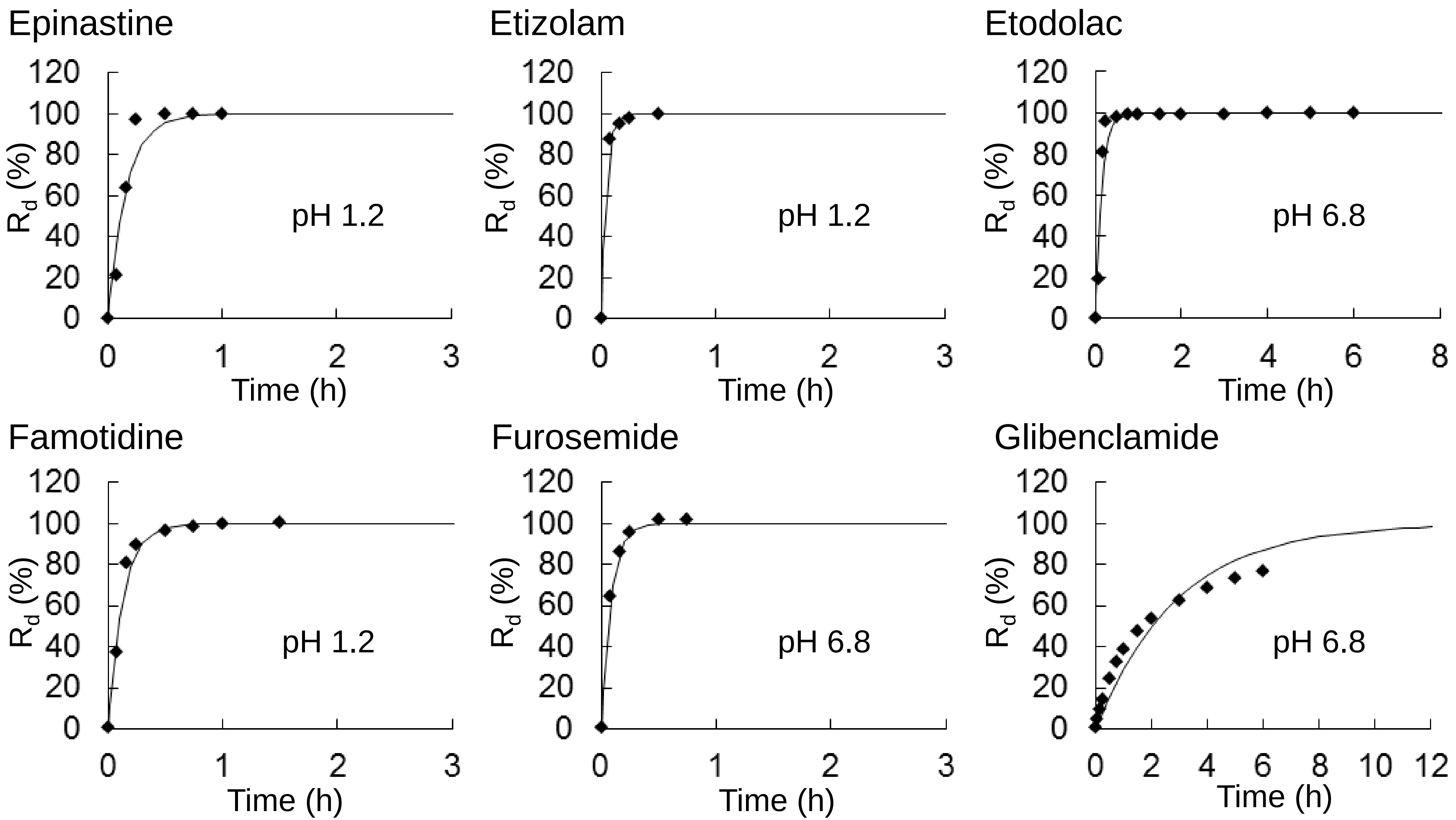
Epinastine
Etizolam
Etodolac
Famotidine
Furosemide
Glibenclamide
pH 1.2
pH 6.8
R_d (%)
Time (h)

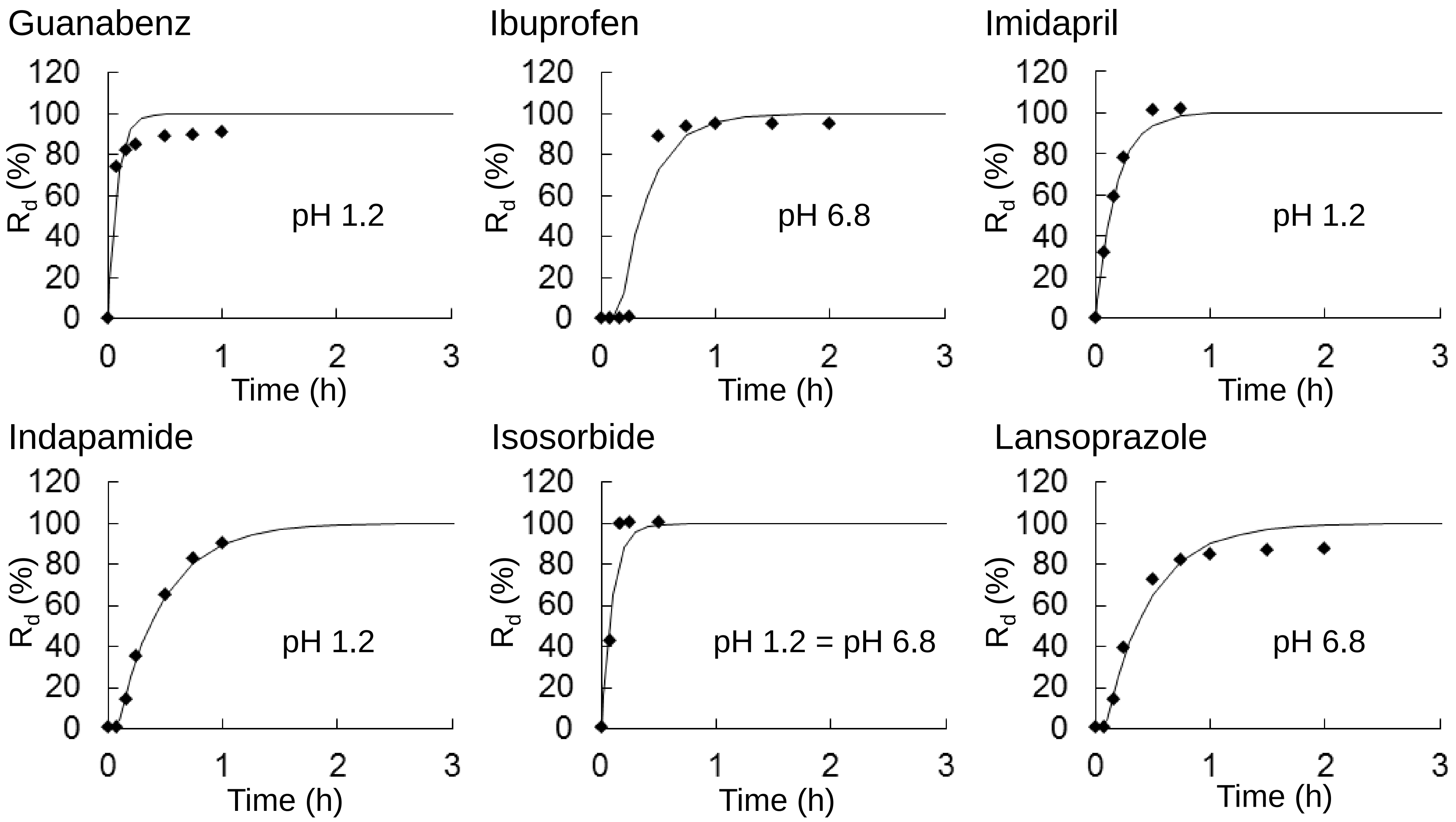
Guanabenz
pH 1.2
Ibuprofen
pH 6.8
Imidapril
pH 1.2
Indapamide
pH 1.2
Isosorbide
pH 1.2 = pH 6.8
Lansoprazole
pH 6.8
R_d (%)
Time (h)
0
20
40
60
80
100
120
0
1
2
3

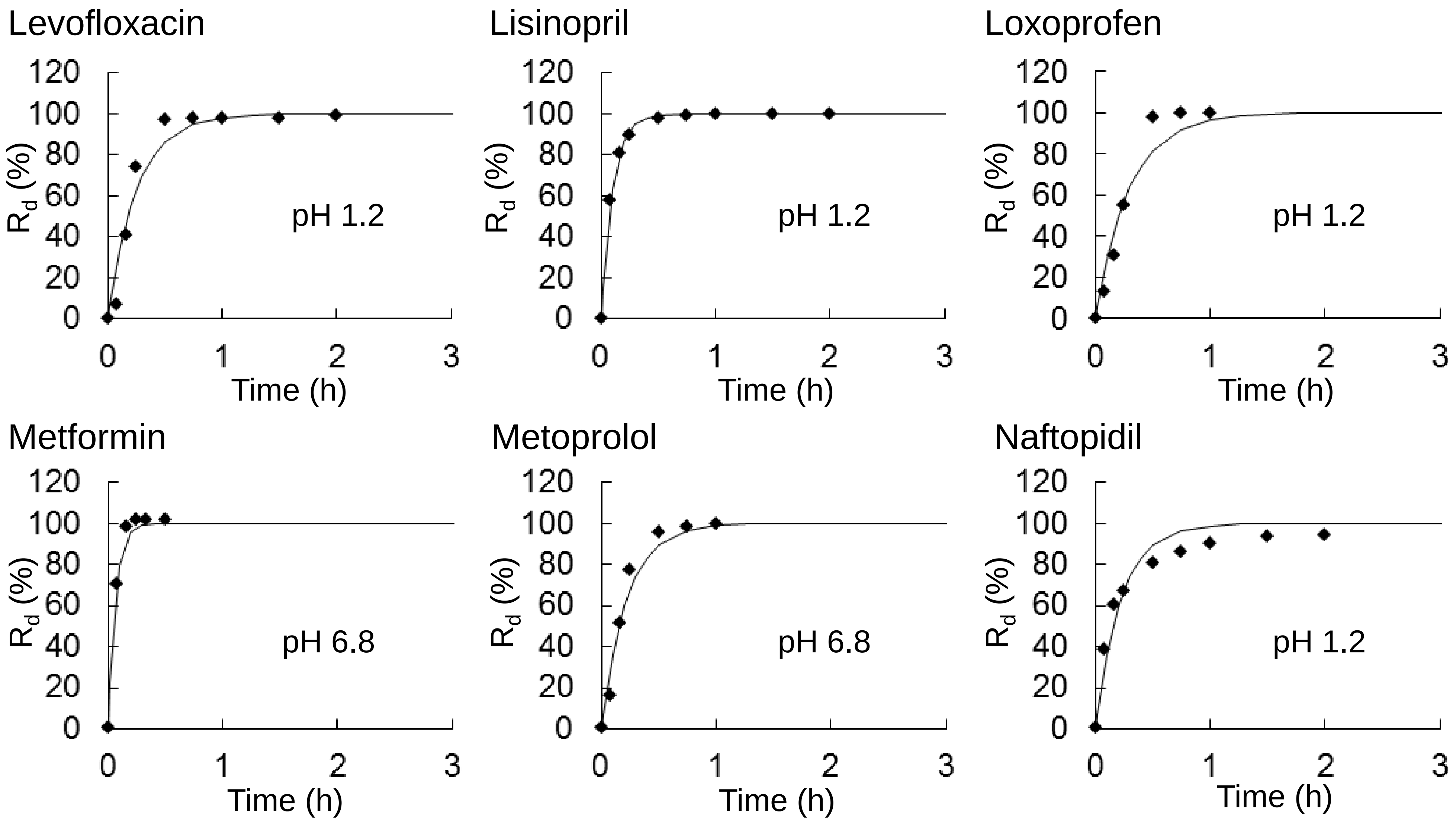
Levofloxacin
Lisinopril
Loxoprofen
Metformin
Metoprolol
Naftopidil
pH 1.2
pH 6.8
R_d (%)
Time (h)
0
20
40
60
80
100
120
1
2
3

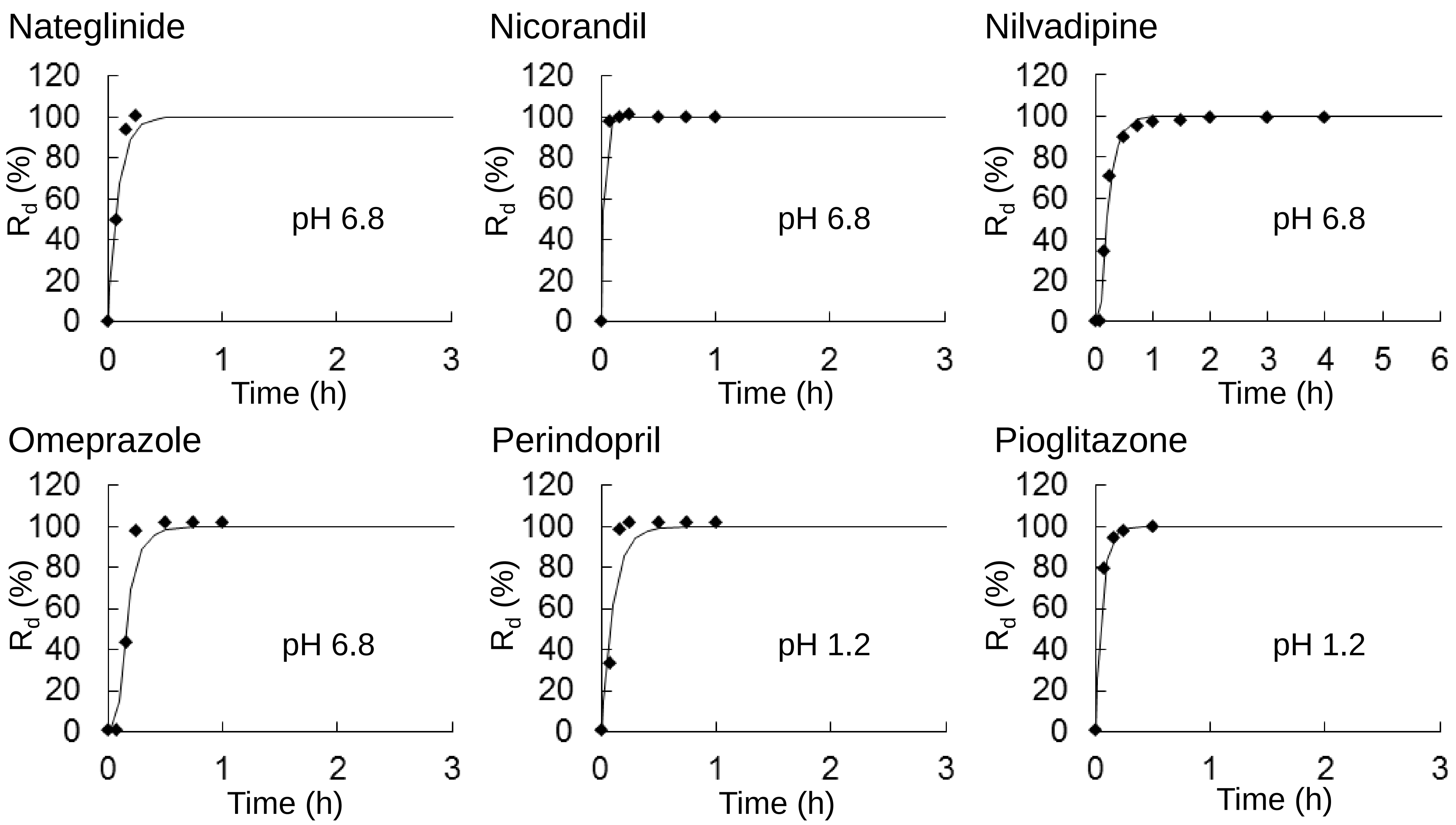

Nateglinide
pH 6.8
Nicorandil
pH 6.8
Nilvadipine
pH 6.8
Omeprazole
pH 6.8
Perindopril
pH 1.2
Pioglitazone
pH 1.2
R_d (%)
Time (h)
0
20
40
60
80
100
120
1
2
3
4
5
6

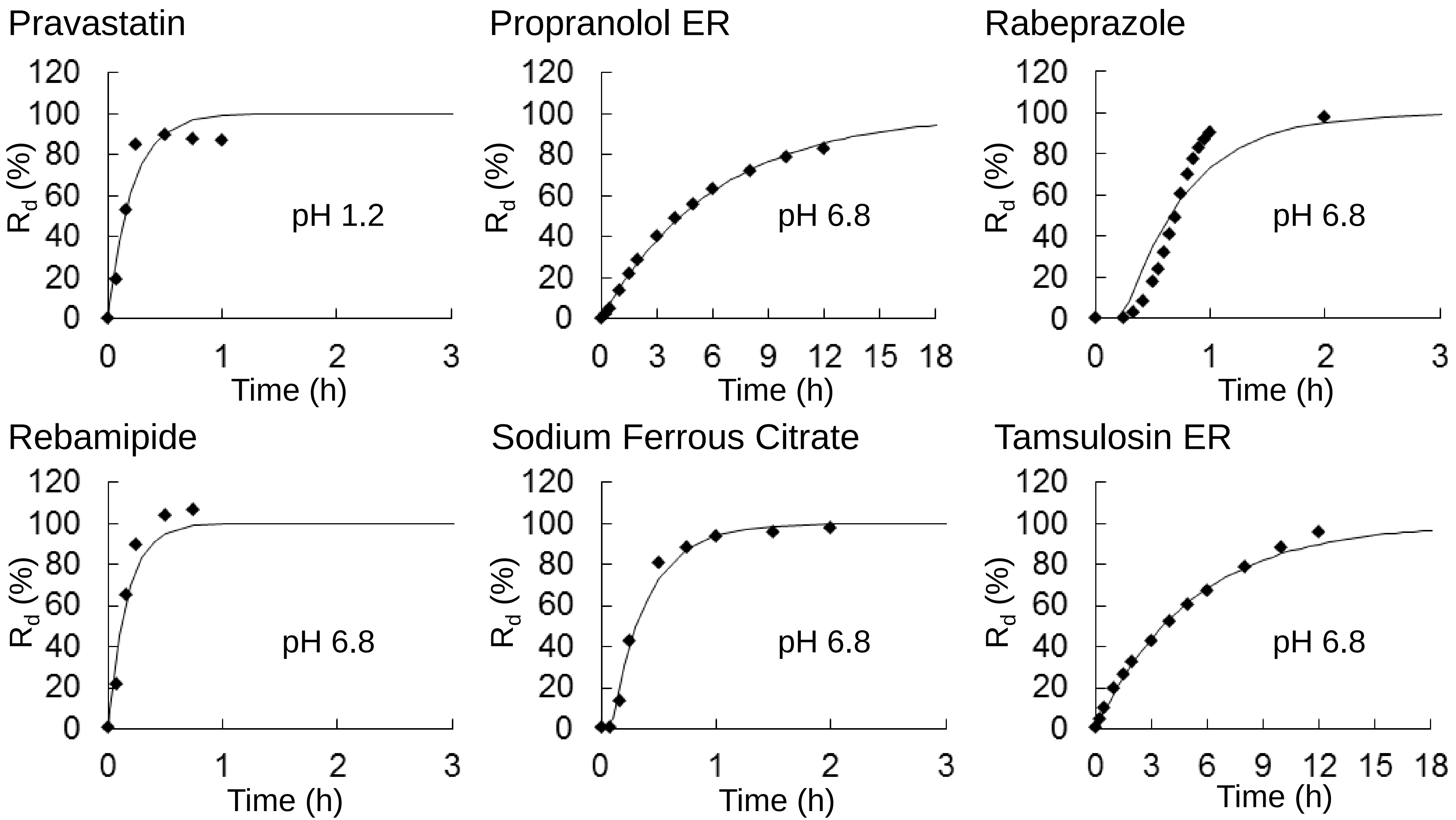

Pravastatin
pH 1.2
Propranolol ER
pH 6.8
Rabeprazole
pH 6.8
Rebamipide
pH 6.8
Sodium Ferrous Citrate
pH 6.8
Tamsulosin ER
pH 6.8
R_d (%)
Time (h)

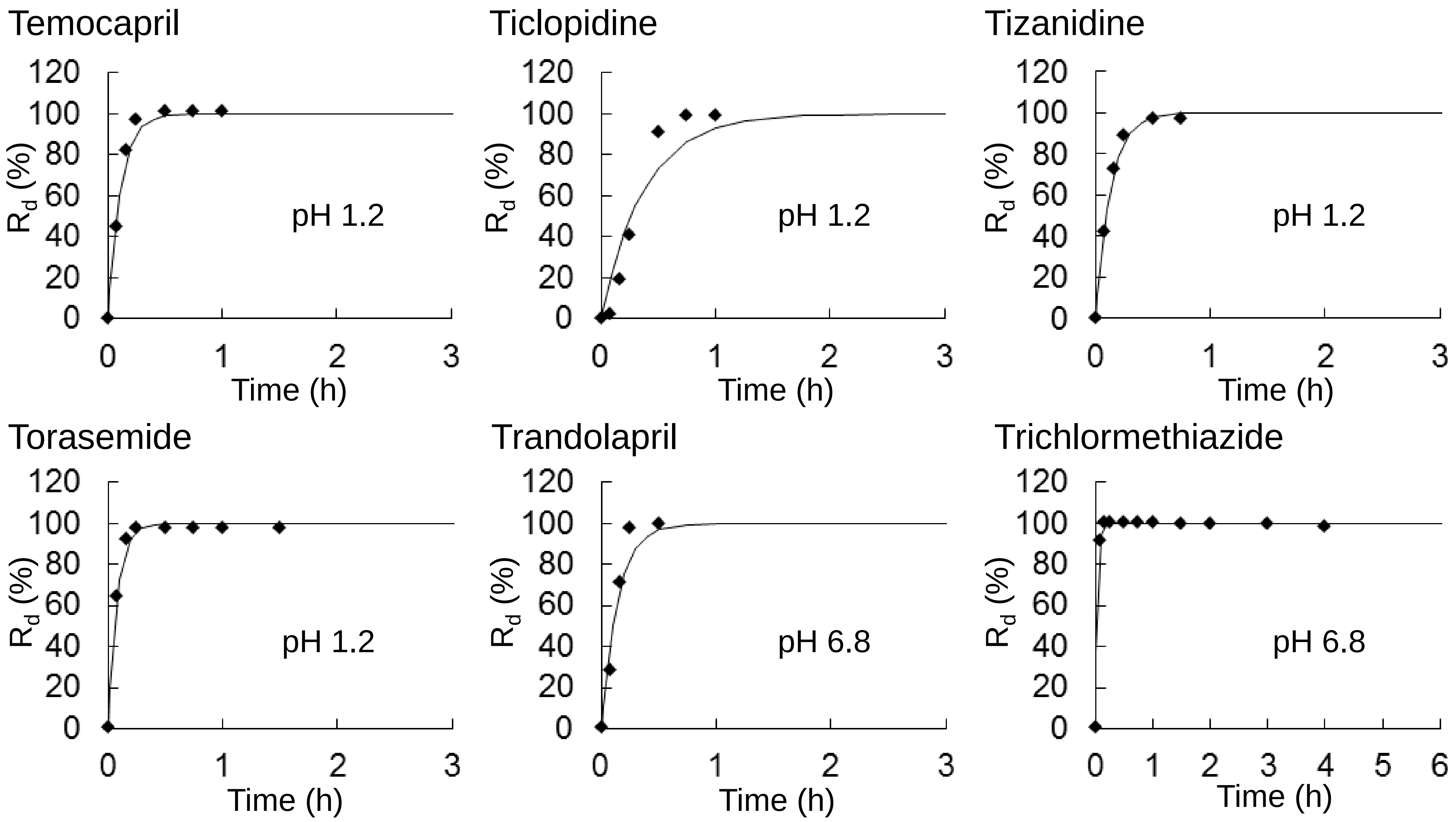

Temocapril
Ticlopidine
Tizanidine
Torasemide
Trandolapril
Trichlormethiazide
R_d (%)
Time (h)
pH 1.2
pH 6.8
0
20
40
60
80
100
120
1
2
3
4
5
6

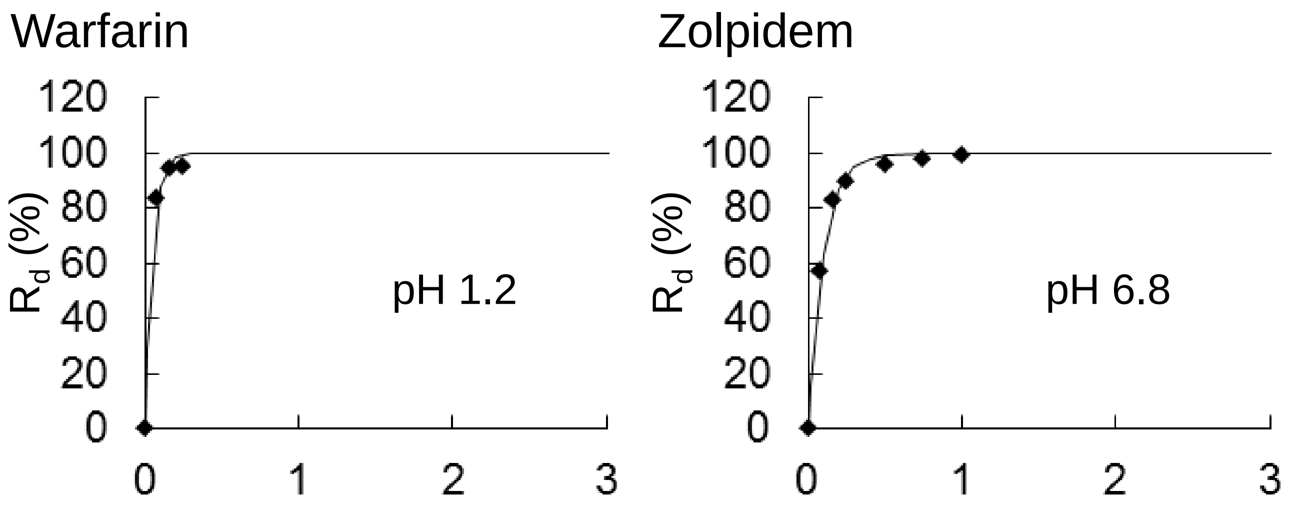

Warfarin
Zolpidem
120
100
80
60
40
20
0
R_d (%)
pH 1.2
pH 6.8
0
1
2
3
